# RETRACTION: Effect of Accelerated Rehabilitation Nursing Programmes on Surgical Site Wound Infection in Patients Undergoing Laparoscopic Cholecystectomy: A Meta‐Analysis

**DOI:** 10.1111/iwj.70262

**Published:** 2025-02-25

## Abstract

**RETRACTION:** WangB. and KouX.‐L. , “Effect of Accelerated Rehabilitation Nursing Programmes on Surgical Site Wound Infection in Patients Undergoing Laparoscopic Cholecystectomy: A Meta‐Analysis,” International Wound Journal 21, no. 1 (2024): e14346, 10.1111/iwj.14346.37592759 PMC10781593

The above article, published online on 17 August 2023, in Wiley Online Library (http://onlinelibrary.wiley.com/), has been retracted by agreement between the journal Editor in Chief, Professor Keith Harding; and John Wiley & Sons Ltd. Following an investigation by the publisher, all parties have concluded that this article was accepted solely on the basis of a compromised peer review process. The editors have therefore decided to retract the article. The authors did not respond to our notice regarding the retraction.

**RETRACTION:** B. Wang and X.‐L. Kou , “Effect of Accelerated Rehabilitation Nursing Programmes on Surgical Site Wound Infection in Patients Undergoing Laparoscopic Cholecystectomy: A Meta‐Analysis,” International Wound Journal 21, no. 1 (2024): e14346, 10.1111/iwj.14346.37592759 PMC10781593

The above article, published online on 17 August 2023, in Wiley Online Library (http://onlinelibrary.wiley.com/), has been retracted by agreement between the journal Editor in Chief, Professor Keith Harding; and John Wiley & Sons Ltd. Following an investigation by the publisher, all parties have concluded that this article was accepted solely on the basis of a compromised peer review process. The editors have therefore decided to retract the article. The authors did not respond to our notice regarding the retraction.

